# New approach to determine the healthy immune variations by combining clustering methods

**DOI:** 10.1038/s41598-021-88272-x

**Published:** 2021-04-26

**Authors:** Claire Liefferinckx, Zacharie De Grève, Jean-François Toubeau, Hélène Perée, Eric Quertinmont, Vjola Tafciu, Charlotte Minsart, Souad Rahmouni, Michel Georges, François Vallée, Denis Franchimont

**Affiliations:** 1 Laboratory of Experimental Gastroenterology, ULB, Brussels, Belgium; 2 Department of Gastroenterology, Hôpital Erasme, ULB, Route de Lennik, 808, 1070 Brussels, Belgium; 3 Electrical Power Engineering Unit, Umons, Mons, Belgium; 4 Unit of Animal Genomics, GIGA-Institute, ULiege, Liège, Belgium

**Keywords:** Computational biology and bioinformatics, Functional clustering

## Abstract

Immune-mediated inflammatory diseases are characterized by variability in disease presentation and severity but studying it is a challenging task. Defining the limits of a healthy immune system is therefore a prior step to capture variability in disease conditions. The goal of this study is to characterize the global immune cell composition along with their influencing factors. Blood samples were collected from 2 independent cohorts of respectively 389 (exploratory) and 208 (replication) healthy subjects. Twelve immune cells were measured in blood together with biological parameters. Three complementary clustering approaches were used to evaluate if variability related to the immune cells could be characterized as clusters or as a continuum. Large coefficients of variation confirmed the inter-individual variability of immune cells. Considering all subset variations in an overall analysis, it appeared that the immune makeup was organized as a continuum through the two cohorts. Some intrinsic and environmental factors affected the inter-individual variability of cells but without unveiling separable groups with similar features. This study provides a framework based on complementary clustering approach for analyzing inter-individual variability of immune cells. Our analyses support the absence of clusters in our two healthy cohorts. Also, our study reports some influence of age, gender, BMI, cortisol, season and CMV infection on immune variability.

## Introduction

The immune system is a key component of human health, and any quantitative or qualitative dysregulation of it can result in infectious diseases, malignancy or immune-mediated inflammatory diseases (IMIDs)^1^. The most common IMIDs are psoriasis, type 1 diabetes, inflammatory bowel diseases or ankylosing spondylitis. These diseases are associated with a significant morbidity and reduce the patient’s quality of life. They also show a large and unpredictable variation in terms of presentation (age at diagnosis, location,…), severity (disease activity and progression) and response to treatment^2^. So far, their clinical management is usually reactive and tailored to their clinical presentation and severity, which does not always allow to reverse tissue damage related to inflammation^3,4^. In that regard, an improved knowledge of the human variation of the immune response could probably help to decipher some predictive variables related to the spectrum of severity of IMIDs, to switch from a reactive management towards a personalized pro-active management that would prevent tissue damage and remodeling related to inflammation^5,6^.

However, studying variations in disease presentation and severity regarding IMIDs is a challenging task due to the high variability of the immune response among these patients. Defining the limits of a healthy immune system is therefore a prior step to capture variability in disease conditions and several healthy cohorts have been published in this perspective^7–10^. While many studies have investigated the inter-individual variability for each immune cell subsets separately, few have profiled the global immune cell composition and their interdependence in healthy cohorts^11–13^. The clustering methods described in the medical literature^11,12^ to capture this immune cell interdependence were mainly based on traditional K-means algorithm which lacks of robustness face to outliers and noise^14^. Likewise, the evaluation metrics used for assessing the quality of the solution given by clustering algorithms were rarely applied, excepted the Silhouette Index^12^. Hence, there is still an open debate on whether the immune system variations should be characterized as discrete groups or as a continuum^15^.

The main goal of our study is to characterize the limits and the overall structure of the immune system variations in a large healthy cohort by leveraging different methods of clustering. In particular, we apply three complementary algorithms, on data gathered from both a base cohort and a replication cohort, and compute adequate metrics to evaluate the relevance of the groups. Our study also aims at analyzing the influence of some intrinsic and environmental factors on the immune composition, which is achieved by ensuring both strict inclusion/exclusion criteria, and a consistent and well-defined study design. We propose in that way a tagged-factor clustering. Hence, the present work aims at presenting a combined clustering framework to better define the inter-individual variability of healthy immune system, which serves as a prior step to better understand the related variability in IMIDs.

## Methods

### Study populations

Strict conditions were applied to build a well characterized and homogeneous cohort. The study cohort (GEOCODE cohort) involved 389 healthy subjects prospectively included between October 2016 and March 2018. The study was approved by the Ethics committee of Erasme Hospital, Brussels, Belgium (Reference number: P2015/425, date approval: 03/11/2015). All used methodologies were in accordance with approved guidelines and were performed in accordance with the Declaration of Helsinki. Each subject signed an informed consent. Inclusion criteria included age between 18 and 65 years, smoking-free status, no use of drugs excepted hormonal contraception or finasteride, benzodiazepine and proton-pump inhibitors, and to be in “good health” (see details in Supplementary methods). Exclusion criteria included participation of another family member to the present study (first–second–third degree), shift workers (chronic jet lag) or any following situations within the previous two weeks of inclusion: actively allergic disease, episode of temperature > 38 °C, dentist consultation or endoscopic operation, vaccination or steroid (systemic or topic) treatment. A second cohort (LIEGE cohort) was used to independently validate clustering results. This replicative cohort involved 208 healthy subjects prospectively included between October 2018 and March 2020. The second cohort was approved by the Ethics committee of CHU Liège, Belgium (Reference number: 2017/214, date approval: October 5, 2017). Again, all methodologies used for the replicative cohort were in accordance with approved guidelines and were performed in accordance with the Declaration of Helsinki. Each subject signed an informed consent.

### Data collection and measurements

A case report form was completed for each subject before collection of blood samples to ensure that inclusion/exclusion criteria were encountered. Gender, age, height, weight, medical and familial history were collected. All blood samples (+ /− 40 ml) were collected between 07h30 and 10h00 a.m. to standardize the circadian cycle after overnight fasting. A fresh EDTA tube was processed within the same day of blood collection for immunophenotyping. Details on immunophenotyping are presented in Supplementary methods. All immune cell counts were expressed by mm^3^. Plasma and sera were aliquoted and stored at − 20 °C for later measurements of cytomegalovirus (CMV)/Epstein–Barr virus (EBV) serologies, C-Reactive Protein (CRP) level, cortisol, estradiol, progesterone, and testosterone. At the end of cohort constitution, all measurements were performed by batch of 20 samples over 4 weeks at the laboratory of Erasme Hospital (LHUB-ULB). IgG CMV, IgG EBV, cortisol, estradiol, progesterone and testosterone were measured using an electro-chemiluminescent immunoassay (Cobas-Roche). EBV and CMV serologies were expressed as U/ml and considered as positive if > 20 U/ml and > 1 U/ml, respectively. Cortisol and testosterone were expressed as nmol/l, while estradiol was expressed as ng/l, and progesterone as μg/l. C-Reactive protein (CRP) level was measured using immunoturbidimetric test (Cobas- Roche), and expressed in mg/l.

### Clustering and tagged-factor clustering

The GEOCODE dataset comprised initially 389 individuals represented by 12 different immune cells, whereas the LIEGE dataset was made of 208 individuals described by 10 different immune cells (consisting in a subset of the 12 GEOCODE different immune cells, see Supplementary methods for the list of considered immune cell subsets). Three complementary clustering methodologies were chosen to strengthen the confidence in the obtained results. *Partition Around Medoids (PAM)*^16^ was firstly applied as it represents a well-known algorithm with some interesting properties (such as, e.g., a better robustness to outliers and noise compared to the traditional K-means algorithm^14^) but it cannot find non-convex clusters. Also, the number of clusters has to be fixed a priori. To alleviate limits related to PAM, density-based clustering, and more particularly the *DBSCAN*^17^ technique, has also been employed. The main idea consists in partitioning the input space by separating dense from sparse zones. The algorithm is in that way able to detect clusters of arbitrary shapes. Finally, *Gaussian Mixture Model (GMM) clustering*^18^, belonging to the family of model-based clustering techniques, was employed in order to ascertain our results.

The two datasets were then augmented by tagging intrinsic and environmental factors. By doing so, we aim at revealing structure in the dataset which could be possibly induced by the consideration of such factors. Briefly, we visually assessed the clustering tendency of a dataset by applying a Principal Component Analysis (PCA)^19^ and t-distributed stochastic neighbor embedding (tSNE)^20^, and we tagged the dataset according to the predefined values of the studied intrinsic/environmental factors. The dataset was first tagged with regards to each factor separately followed by a multi-factor tagging in which all possible combinations of intrinsic/environmental factors were tested. In total, 31 combinations were investigated. This tagged-factor clustering was developed to visually unveil the potential presence of substructure in the GEOCODE cohort even though first clustering methods suggest a one cluster solution.

### Models calibration and evaluation methods

Evaluating partitions obtained by clustering approaches faces two main challenges. First, the ground truth is usually non-accessible to assess the quality of a partition, so that the ‘true’ number of clusters is unknown in practice. Second, the complete absence of structure in a dataset, which corresponds to a situation where a single cluster solution is the most suited, is not easily quantified by common clustering evaluation methods, especially in the case of partitional algorithms. To deal with these two challenges, three different evaluation metrics were employed for assessing the quality of the solution given by the partitional clustering algorithms (the PAM algorithm in this case) and infer the correct number of clusters, i.e. the *Silhouette Index*, the *Gap Statistic*, as well as a Cluster Stability Analysis. On the other hand, an ad-hoc evaluation criterion was provided for the DBSCAN and GMM clustering techniques, since these approaches are inherently able to suggest the best number of clusters. More details regarding the data pre-processing (including details on the management of missing data), the clustering methodologies, and model calibration and evaluation methods are presented in Supplementary Methods. Clustering analyses, evaluation methods and associated graphs were performed using R Software version 3.6.1 (R Core Team (2020). R: A language and environment for statistical computing. R Foundation for Statistical Computing, Vienna, Austria. URL https://www.R-project.org/).

### Statistical analysis

Mann–Whitney test was used for comparing univariate distributions of non-parametric and continuous variables. Results were therefore expressed as median with interquartile range (IQR 25–75). An univariate general linear model was used to analyze the variance of each leukocyte subset (dependent variable) by taking into account of independent variables such as sex, use of pill, age and BMI). Bonferroni correction was used for correcting multiple testing. Also, independent variables were integrated in linear regression and the coefficient of determination (R^2^) was calculated for each independent variable. This coefficient reflects the proportion of the variance in the dependent variable that is predictable from the independent variables. Pearson χ^2^ test was used to compare categorical variables. Pairwise Spearman's rank correlation coefficient was used for quantifying correlation between non-parametric variables. Significant difference was set for *p*-value < 0.05. The analyses were performed with SPSS version 20.0 (SPSS Inc., Chicago, IL, USA).

## Results

### Study cohort and related characteristics

The GEOCODE cohort, the main study cohort, included 389 individuals distributed as 237 females and 152 males. The median age (29 years, IQR 25–39) was similar regardless of gender but BMI was higher in males (*p* < 0.0001). Seven percent (n = 26) of subjects were not of European origin. A familial history of IMIDs was found in 5.7% (n = 22) while a personal history of allergy was found in 4.1% (n = 16). Among females, 66% (n = 157) reported contraceptive pill use and 5 menopausal females used hormonal substitution. Aside from contraceptive pill, a total of 3.3% (n = 13) of the subjects were taking authorized drugs (See Supplementary methods). None of the subjects was active smoker. Serology for EBV and CMV were positive in 90.1% (n = 345/383) and 53.9% (n = 207/384), respectively.

### Inter-individual variability of the immune composition in unstimulated conditions

**Figure 1:**
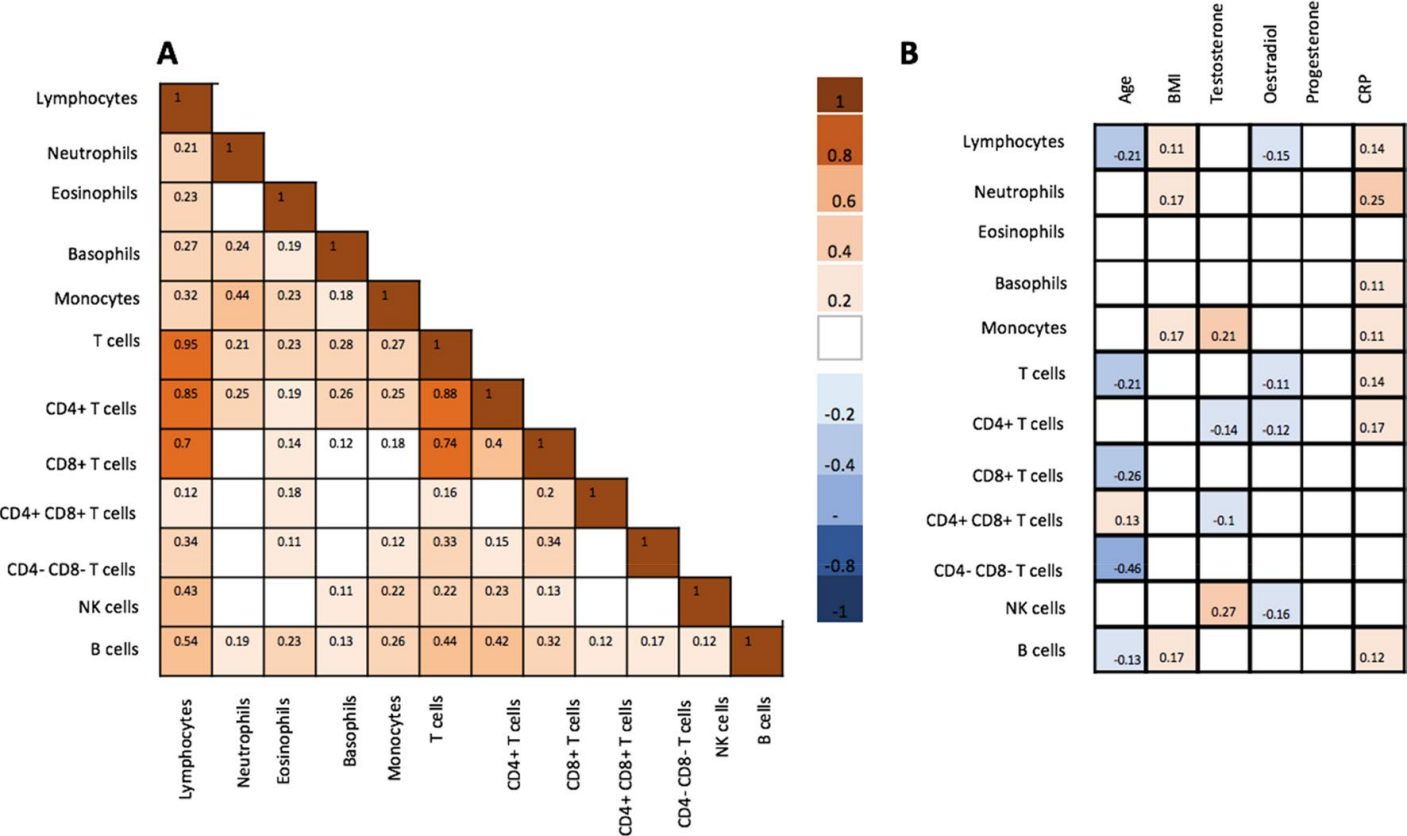
Correlation of baseline immunological parameters and phenotype (age, BMI) at baseline. (**A**) Correlation between the all leukocyte subsets. (**B**). Correlation between the all leukocyte subsets and phenotypic characteristics such as age, BMI, hormones and CRP. Spearman’s rank correlation was used. If the correlation was not significant, a blank square is shown. Colored squares indicate when a correlation was significant (*p* < 0.05).

Regarding cell count for each leukocyte subset, a large inter-individual variability was observed with a coefficient of variation ranging from 30 to 100% within the two cohorts (Supplementary Fig. 1). In the specific case of basophil count in GEOCODE cohort, the coefficient of variation has to be interpreted with regards to methodology (See Supplementary methods). Likewise, although the normal CD4/CD8 ratio for a healthy subject is believed to be around 2, CD4/CD8 ratios observed in our cohort exposed a large range between 0.1 and 12.4. Multiple positive correlations between each two leukocyte distributions were observed (Fig. 1A). With age and BMI as covariates, monocyte and NK cells counts were higher in males while T cells (including CD4 + T cells) were higher in females (Table 1). However, after additional correction for contraceptive pill use, only monocyte and NK cell distributions remained influenced by gender (Table 2). Considering females, only CD4 + T cells counts was significantly higher with use of contraceptive pill (Table 3).

**Table 1:** Leukocyte proportions according to gender with age and BMI in covariates.

	Female (n = 237)	Male (n = 152)	*p*-value
Neutrophils	3100 (2500–3850)	3000 (2500–3700)	0.17
Lymphocytes	1866 (1586–2231)	1830 (1495–2158)	0.32
Basophiles	0 (0–0)	0 (0–0)	**–**
Eosinophils	100 (100–200)	100 (100–200)	0.25
Monocytes	400 (400–500)	500 (400–600)	**< 0.001**
T cells	1483 (1185–1772)	1365 (1073–1644)	**0.012**
CD4 + T cells	912 (744–1154)	786 (645–998)	**< 0.001**
CD8 + T cells	456 (362–588)	466 (328–602)	0.87
CD4 + CD8 + T cells	5 (3–14)	3 (2–13)	0.85
CD4-CD8- T cells	48 (31–76)	52 (32–82)	0.24
NK cells	175 (124–247)	228 (174–331)	**< 0.001**
B cells	180 (130–242)	188 (130–256)	0.65

*p*-values are highlighted in bold when significant

**Table 2:** Leukocyte proportions between males and females not using contraceptive pill with age and BMI in covariates.

	Male (n = 152)	Female w/o use (n = 77)	*p*-value
Neutrophils	3000 (2500–3700)	3100 (2500–3800)	1
Lymphocytes	1830 (1495–2158)	1781 (1488–2080)	1
Basophiles	0 (0–0)	0 (0–0)	–
Eosinophils	100 (100–200)	100 (100–200)	1
Monocytes	500 (400–600)	400 (400–500)	**0.025**
T cells	1365 (1073–1644)	1437 (1071–1657)	1
CD4 + T cells	786 (645–998)	813 (677–1069)	1
CD8 + T cells	466 (328–602)	416 (300–543)	0.6
CD4 + CD8 + T cells	3 (2–13)	5 (3–14)	1
CD4-CD8- T cells	52 (32–82)	44 (30–67)	1
NK cells	228 (174–331)	178 (131–249)	**0.001**
B cells	188 (130–256)	175 (116–239)	1

*p*-values are highlighted in bold when significant

**Table 3:** Leukocyte proportions among females using or not contraceptive pill with age and BMI in covariates.

	Use (n = 171)	No use (n = 77)	*p*-value
Neutrophils	3200 (2500–4000)	3100 (2500–3800)	1
Lymphocytes	1931 (1647–2350)	1781 (1488–2080)	0.22
Basophiles	0 (0–0)	0 (0–0)	–
Eosinophils	100 (100–200)	100 (100–200)	0.67
Monocytes	400 (400–500)	400 (400–500)	1
T cells	1518 (1278–1849)	1437 (1071–1657)	0.92
CD4 + T cells	941 (772–1171)	813 (677–1069)	**0.03**
CD8 + T cells	500 (386–616)	416 (300–543)	0.53
CD4 + CD8 + T cells	5 (3–14)	5 (3–14)	1
CD4-CD8- T cells	53 (32–84)	44 (30–67)	1
NK cells	173 (118–247)	178 (131–249)	1
B cells	186 (139–243)	175 (116–239)	1

*p*-values are highlighted in bold when significant

### The inter-individual variability of circulating immune cells is organized as a continuum

**Figure 2:**
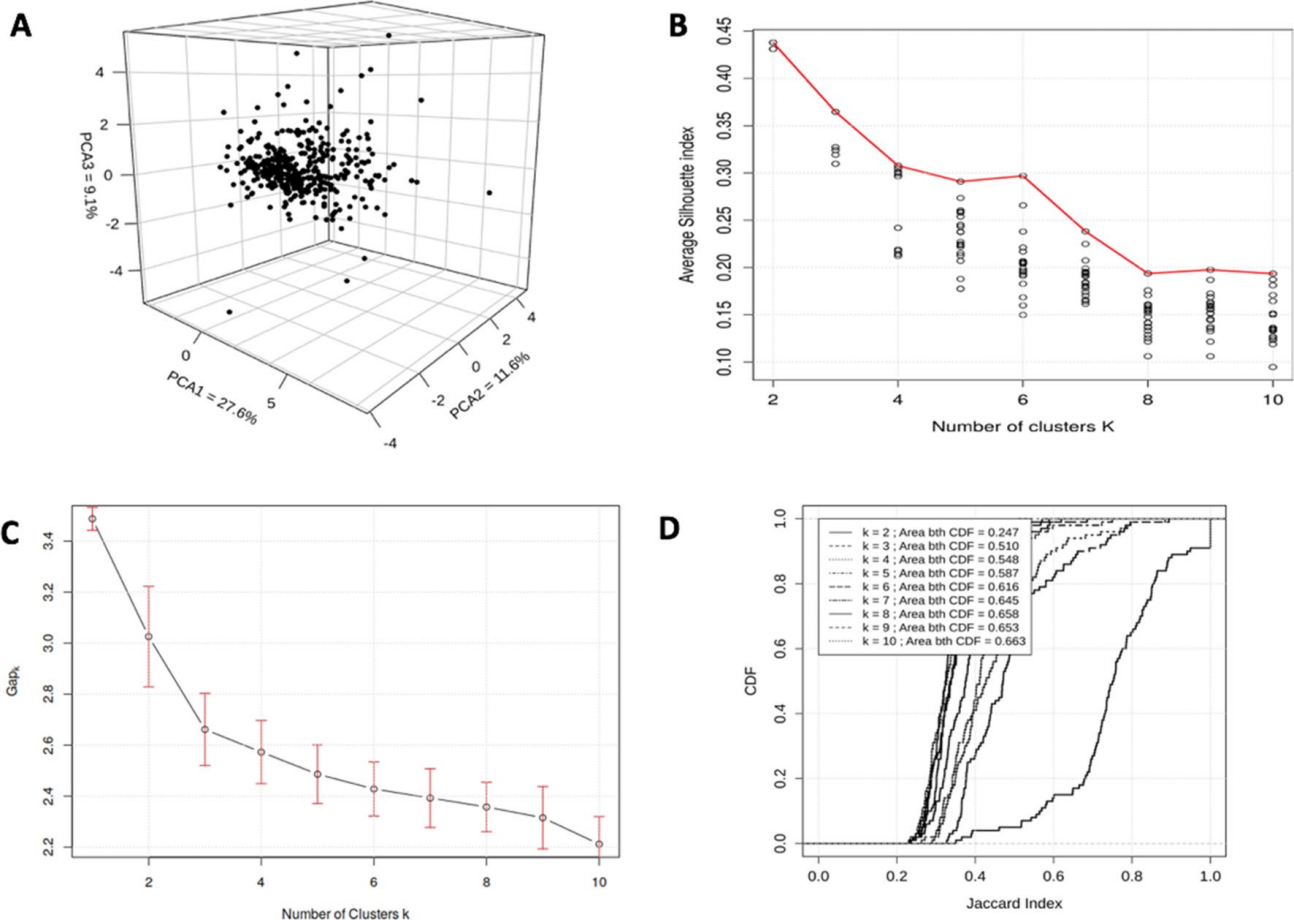
Clustering approaches applied to GEOCODE study cohort. (**A**) Three-dimensional PCA of the study was used as a qualitative visual criterion. (**B**) The graph shows the average silhouette indices obtained by running PAM with an increasing number of clusters *K*. For each value of *K*, 20 instances of the PAM algorithm were run with a random initialization of centroids, in order to mitigate the effect of local minima. The corresponding solutions are represented by black circles, whereas the best solutions for each *K*, i.e. the partitions which maximize the Silhouette Index, are connected by a plain red line. (**C**) The graph shows the Gap Statistic as a function of the number of clusters. For each value of *K*, the Gap Statistic was computed by comparing the clustering solution obtained on the dataset with partitions extracted from 200 unstructured datasets randomly generated. (**D**) The graph shows the results of the Cluster Stability Analysis, for which 100 random subsamples of the original dataset have been clustered and compared using the Jaccard Index. Graphs were performed using R Software version 3.6.1

The first 3 Principal Components from the PCA of the study cohort allowed to depict 48.3% of the overall variability (Fig. 2A) but no particular tendency in terms of structure was observed using that qualitative visual criterion. We confirmed that first hypothesis quantitatively by applying clustering techniques on the whole 12-dimensional study cohort, and by interpreting the corresponding evaluation metrics. Figure 2B shows the average Silhouette Indices obtained by running PAM with an increasing number of clusters *K*. It is shown that decreasing the number of clusters leads to higher (and thus better) Silhouette Indexes, thus suggesting that the data cannot be clustered (since even the quality of the two-cluster solution is quite low). However, Silhouette index is by definition not able to confirm a lack of structure in a given dataset. In that regard, two other metrics were computed on the PAM results in order to confirm a possible single-component structure. Figure 2C shows the Gap Statistic as a function of the number of clusters. Interestingly, a solution with one single cluster, which maximizes the Gap Statistic, was suggested in this case. Figure 2D depicts the results of the Cluster Stability Analysis, for which 100 random subsamples of the original dataset have been clustered and compared using the Jaccard Index. According to Ben-Hur et al.^21^, the correct number of clusters can be found by identifying the CDF with the most pronounced step. Since such a step was not observed in our results, the Cluster Stability Analysis also suggested a single component structure for the GEOCODE cohort.

A second and independent cohort, namely LIEGE cohort, was used to confirm this single cluster solution **(**Supplementary Fig. 2). The phenotypic characteristics of this second cohort were compiled in Supplementary Table 1.

**Figure 3:**
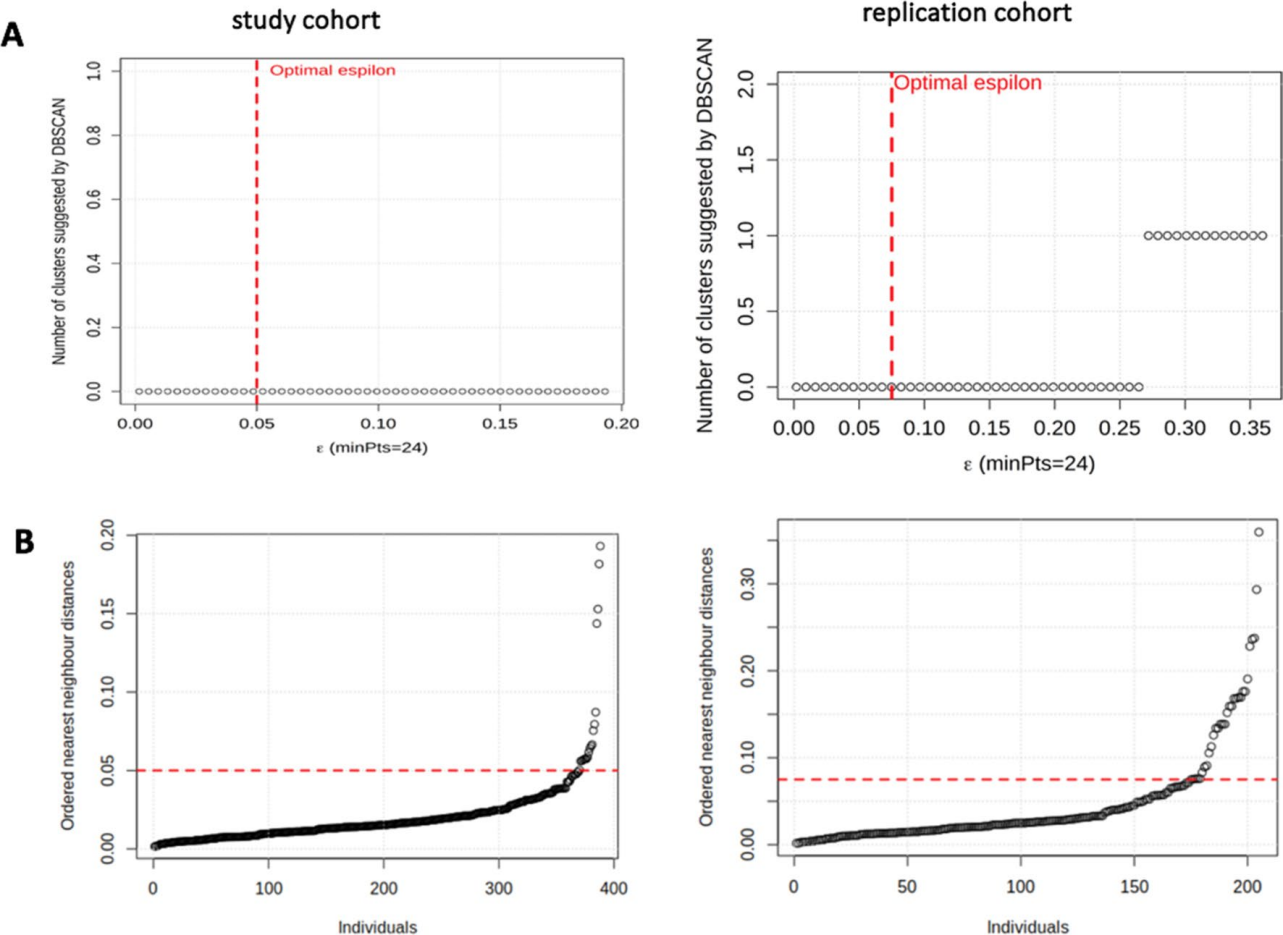
DBSCAN clustering approach. (**A**) Graphs suggested that the data objects were noisy points for the two cohorts, since the obtained number of clusters at the optimal value of *ε* is zero. (**B**) Graphs show that individuals tend to be closely aggregated together. Graphs were performed using R Software version 3.6.1

The two other clustering approaches, namely DBSCAN (Fig. 3) and GMM clustering (see Supplementary Fig. 3), both confirmed this single cluster structure with both GEOCODE and LIEGE cohorts, thereby showing the consensus in terms of number of clusters between PAM, DBSCAN and GMM clustering. It should be noted that, more generally and if needed, partitions obtained by using different clustering algorithms may be combined into a robust consensus clustering result using other approaches as described in the literature^22,23^. The distances of each object to its nearest neighbor sorted in ascending order further demonstrated that individuals tend to be closely aggregated together, in both cohorts. Even if several individuals were more distant from the others, they do not represent a convincing well-defined separate cluster (Fig. 3).

### Effects of intrinsic and environmental factors on the inter-individual variability of the immune composition

**Figure 4:**
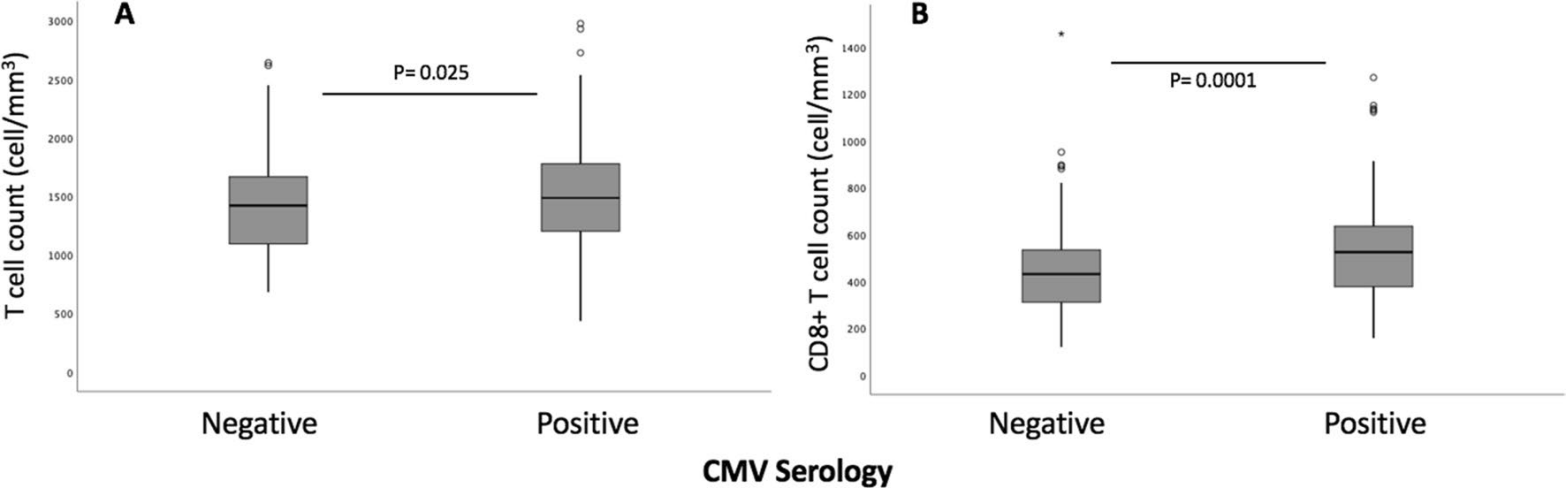
Distributions of immune cells according to CMV serology. (**A**) T cell counts according to CMV serology (n = 207 seropositive subjects, n = 177 seronegative subjects). (**B**) CD8 + T cell and CD4-CD8- T cell counts according to CMV serology (n = 207 seropositive subjects, n = 177 seronegative subjects). Graphs were performed using SPSS version 20.0

Intrinsic factors were age, gender, hormones (testosterone, progesterone, estradiol and cortisol), BMI and history of allergy while environmental factors were season at inclusion, use of contraceptive pill and, EBV and CMV serologies. Estradiol was negatively correlated with several leukocyte subsets, i.e., lymphocytes, CD4 + T cells, and NK cells. Testosterone was positively correlated with monocytes and NK cells, and negatively correlated with CD4 + T cells. No correlation was found between immune cells and progesterone. While the median age was 29 years, age was negatively correlated with all lymphocyte distributions, excepted CD4 + T cells and CD4 + CD8 + T cells. In this latter case, CD4 + CD8 + T cells were positively associated with age (Fig. 1B). Although no subjects with acute allergies were included, eosinophil count was higher in subjects with allergic history, *p* = 0.009 (median: 200 (IQR 100–350) vs. median 100 (IQR 100–200)). Positive EBV serology had no influence on leukocyte distributions. On the contrary, impact of CMV status on lymphocyte repertoire was significant even with age was considered as covariate. A positive CMV serology was associated with a higher median for T cells (median 1485, IQR (1196–1778) vs. median 1419 IQR (1085–1671), *p* = 0.025) and CD8 + T cells (median 525, IQR (377–638) vs. median 432 IQR (311–533), *p* = 0.0001) (Fig. 4).

BMI and cortisol represented the two main factors explaining variance of the different circulating immune cells. However, depending of the leukocyte subset, the type of factors and their influence vary. For instance, the history of allergy represents the best factor explaining eosinophil variance while gender is the best factor explaining NK cell variance. The influence of seasons on immune cell variance was marginal for all leukocyte subsets but CD4 + CD8 + T cells. In this latter case, the level of CD4 + CD8 + T cells was significantly higher during Autumn and Winter before decreasing during Spring and Summer. Nonetheless, all available factors jointly considered were found to explain less than 20% of the overall variance of the different circulating immune cells (Supplementary Fig. 4).

### The combined effect of factors does not induce the emergence of clusters

**Figure 5:**
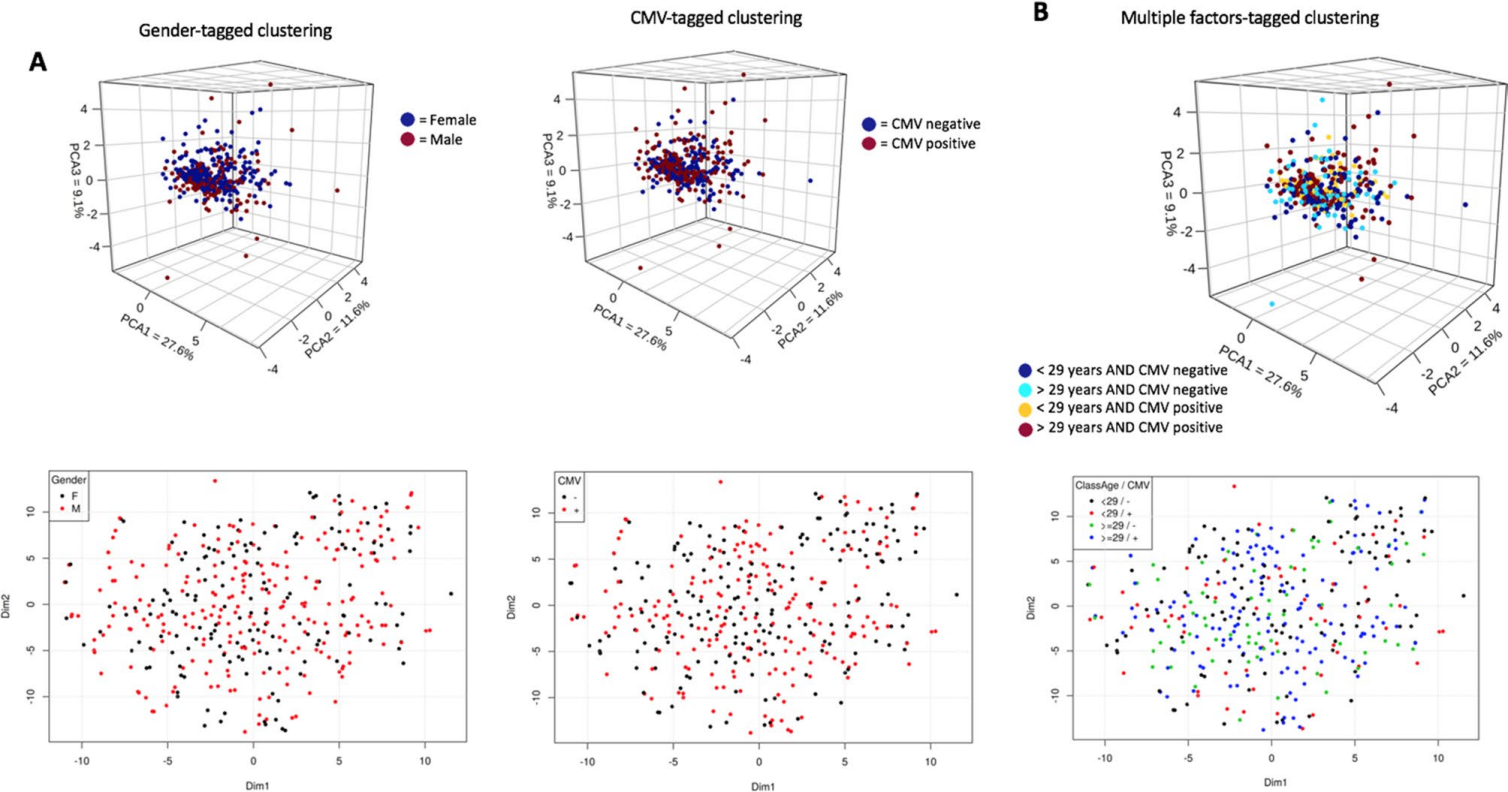
Three-dimensional PCAs of the GEOCODE study cohort with intrinsic and extrinsic factors. (A) Three dimensional PCAs and tSNE of the GEOCODE study cohort are depicted with tagged individuals according to age, gender, EBV/CMV status separately. **(B**) Three-dimensional PCA and tSNE of the GEOCODE study cohort is depicted with tagged individuals according to combination of the age and CMV factors. Graphs were performed using R Software version 3.6.1

Although the overall structure of circulating immune cells was organized as a continuum, the impact of intrinsic factors (gender, age and BMI) or extrinsic factor (CMV/EBV status) could unveil a sub-structure with emergence of discrete groups. First, the influence of factor was analyzed by tagging the dataset with each intrinsic and extrinsic factor separately. Second, all possible combinations of intrinsic/environmental factors were tested by a multi-factor tagging (see “Methods” section). From the 31 resulting combinations, none allowed discovering the emergence of discrete groups. Figure 5A illustrates 3-dimensional PCAs of the study cohort as well as tSNE with tagged individuals according to age, gender, EBV/CMV status separately. No structure tends to visually appear. Interestingly, the median age of the replicative cohort was higher than the study cohort (29 years vs. 51 years). Despite this median older age in the replicative cohort, a substructure did not appear in this case as well. Figure 5B combines the age and CMV factors in a 3-dimensional PCA and tSNA. Again, no shift in immune variability seems to appear.

## Discussion

This study provides a methodological framework for capturing the inter-individual variability of the circulating immune cells in a significant cohort of healthy subjects and the inter-relationships between these immune cells. By leveraging complementary clustering methods, we observed that the integrated variabilities of circulating immune cells are organized as a continuum since no clusters could be identified. A second and independent cohort was used to confirm the results, thus ensuring the robustness of our methodology. Moreover, a tagged-factor clustering based on intrinsic/environmental factors (gender, age, BMI, EBV and CMV status) did not allow to unveil the emergence of clusters. Interestingly, aside from clustering analyses, we reported that gender (and related hormones), BMI, cortisol, EBV/CMV status and seasons have some influence immune variations. Also, we observed that gender difference in CD4 + T cells seemed more related to the use of contraceptive pill than gender itself. Overall, less than 20% of the overall variability of immune cells was explained by these intrinsic and environmental factors.

So far in the literature, the inter-individual variability of immune cells has been mainly analyzed for each immune subset separately, which prevented to capture the complexity of inter-relationships between immune subsets and thus to have a global understanding of human immune system variation^12,15^. This question is however important to properly define individuals with outlier measurements, not only for one leukocyte subset but for the combination of all of them. Through a robust clustering methodology including three complementary approaches, we observed that the leukocyte subsets were organized as a continuum and we replicated these observations in an independent cohort. This absence of clusters of individuals sharing similar variation of immune cell composition is aligned with recent findings^12,13^. Likewise, the homogenous blood cell composition observed in our cohorts reflects a balanced homeostasis expected in healthy subjects. As gender, age, CMV status contribute to shape the immune inter-individual variability^7,24–27^, we integrated these factors (alone and in a combinatorial analysis) to ensure that clusters do not emerge in some specific situations. Overall, our results give more arguments to reasonably exclude, in healthy subjects, the existence of discrete groups including individuals with similar variation of immune cell composition.

Several recent studies have described the large variability of the immune system in healthy subjects^7–10^. These studies, including ours, respond to the need of defining the boundaries of healthy immune system^10^ and represent a precondition before capturing inter-individual variability of IMIDs. Overall, numerous intrinsic and extrinsic factors contribute to explain the observed variability between healthy individuals. By contrast to other healthy cohorts, we applied stricter inclusion/exclusion criteria to rule out the impact of well-known factors on this variability^10,28^. For instance, an active smoking status was prohibited as it is well described that tobacco largely influences immune cell frequencies^29^. Likewise, the use of almost all drugs was prohibited (see “Methods” section). However, due to the frequency of contraceptive pill use in healthy women, this drug was accepted and we took opportunity of analyzing how contraceptive pill can impact the immune variability. This hormonal drug seems to significantly impact the lymphocytes and most specifically T cells. Interestingly, the gender difference in CD4 + T cells, recently reported^27^, disappeared when females not using contraceptive pill were compared to males which may suggest that the contraceptive pill could impact CD4 + T cells. Indeed, we observed that use of contraceptive pill was associated with a higher absolute number of circulating CD4 + T cells. However, due to the complex influence of hormones (both endogenous and synthetic) on immune system, it is difficult to known whether these higher levels of circulating CD4 + T cells actually represent a susceptibility risk for the development of IMIDs^30,31^.

CMV is known to largely influence T cells^32^ but its impact (alone or with other factors) on the inter-relationships of immune subsets has been less explored. As seropositive CMV status combined with age have been associated with shifts in immunotypes^12^, we explored the combined effect of these factors on the emergence of discrete groups but we did not unveil clusters. By contrast to CMV, influence of EBV on immune cell frequencies has not been described in the literature. One explanation could be the very high prevalence of EBV in populations, making it difficult to compare seropositive and seronegative individuals. While the median age of our study was quite young, i.e., 29 years, EBV serology was positive in 90.1% of cases which is in line with reported epidemiologic data^33^.

The proposed study has some limitations which can be discussed. Firstly, due to our strict criteria of inclusion, our study cohort is characterized by a narrowed range of age which precluded any evaluation of the influence of immune senescence on the emergence of clusters over time. Likewise, the opposite correlations between age and, CD4 + T cell and CD8 + T cell subsets appeared contradictory. However, it is described in the literature that CD4 + T cells and CD8 + T cells behave differently in response to aging^34^. This dynamic behavior has not been captured due to the young median age of our GEOCODE cohort and could explain this apparent discrepancy. Nonetheless, the older age of individuals in the replicative cohort allowed us to partly exclude the involvement of age on a potential shift. Secondly, our analysis on inter-relationships between immune cells included 12 leukocyte subsets, which represents only a small part of all circulating leukocyte subsets. We therefore cannot exclude that the addition of other leukocyte subsets unveils clusters. However, it should be noted that others research teams did not find clusters despite the use of such additional leukocyte subsets^12,13^. Thirdly, a significant part of the overall variability could be explained by the genetic makeup of individuals^9,26,35^ and the influence of microbiota^25,36^, but these factors were not available in this study. Finally, our data was restricted to analyze the variations of leukocyte subset distributions. In that regard, the variations in the transcriptional and translational profiles of leukocyte subsets as well as variations in the functional capacity in response to immunological stimulation were not investigated.

On the other hand, several strengths can be highlighted. Firstly, the study cohort had significant size, i.e., 389 healthy subjects, with very few missing data (only few CMV/EBV serologies were missing) and based on strict criteria of inclusion and exclusion, which gave high confidence in the quality of data and precluded any substantial influence of external factors on the immune inter-individual variability such as the presence of a latent infection, a latent IMID or the influence of drugs. Secondly, a strict standardization was applied for immune cells measurements (See Supplementary methods) which reduced technical variability. Finally, the absence of discrete groups in clustering analysis remains a challenging point because it is difficult to firmly prove it (See “Methods” section). However, the use of three complementary clustering approaches in combination with relevant evaluation metrics, i.e., silhouette index, gap statistic, cluster stability analysis and qualitative visual human evaluation, alleviate this issue and strengthen our results. In addition, we replicated our results in a second and independent (LIEGE) cohort.

In conclusion, our study provides a framework based on complementary clustering approach for analyzing inter-individual variability of immune cells and confirms the absence of clusters in our two healthy cohorts. Also, our study reports some influence of age, gender, BMI, cortisol, season and CMV/EBV infections on the distribution of peripheral immune cells.

## Supplementary information

Supplementary Information.

## Abbreviations

BMI: Body mass index
CMV: Cytomegalovirus
CRP: C-reactive protein
DBSCAN: Density-based clustering
EBV: Epstein–Barr virus
GMM: Gaussian mixture model
IMIDs: Immune-mediated inflammatory diseases
PAM: Partition around medoids
PCA: Principal component analysis
tSNE: t-Distributed stochastic neighbour embedding
