# Supplementary material for: New approach to determine the healthy immune variations by combining clustering methods
Source: Sci Rep. 2021 Apr 26;11:8917. doi: 10.1038/s41598-021-88272-x (PMC8076194; doi:10.1038/s41598-021-88272-x)

<u>Supplementary information file</u>

Towards identifying the healthy immune cell organization using clustering approaches

Claire Liefferinckx[1,2*], Zacharie De Grève[3], Jean-François Toubeau[3], Hélène Perée[4], Eric Quertinmont[1], Vjola Tafciu[1], Charlotte Minsart [1,2], Souad Rahmouni[4], Michel Georges[4], François Vallée[3] and Denis Franchimont[1,2]

1, Laboratory of experimental Gastroenterology, ULB, Brussels, Belgium
2, Department of Gastroenterology, Hôpital Erasme, ULB, Brussels, Belgium
3, Electrical power engineering unit, Umons, Mons, Belgium
4, Unit of animal Genomics, GIGA-Institute, ULiege, Liège, Belgium

***Corresponding author**:

Claire Liefferinckx, MD, PhD
Department of Gastroenterology, Hopital Erasme, ULB, Brussels, Belgium
Route de Lennik, 808
1070 Brussels
Belgium
claire.liefferinckx@erasme.ulb.ac.be

Supplementary methods

Inclusion/exclusion criteria for GEOCODE study Cohort

Inclusion criteria were:

- Age between 18 and 65 years
- Smoking-free status. A subject with an history of smoking use was included if stop > 2 months
- No drug allowed except:
    - Hormonal contraception
    - Finasteride
    - Benzodiazepine
    - Proton-pump inhibitors
- To be in "good health" (See table here below)

**Prohibited conditions**	**Allowed conditions**
• IMIDs<br>• Allergic disease in exacerbation<br>• Chronic viral disease such as HBV, HIV, HCV<br>• Chronic endocrinologic disease<br>• Chronic vascular disease<br>• Chronic respiratory disease<br>• Chronic neurologic disease<br>• Chronic gastroenterological disease<br>• Chronic renal disease<br>• Chronic articular disease<br>• Oncologic disease (<5 years)<br>• Surgery* (< 4 weeks)<br><br>*If surgery was associated with disease resolution	• Reflux<br>• Osteoarthritis<br>• Cataract<br>• Anxiety

Exclusion criteria were:

- Age <18 or > 65 years

- Positive smoking status
- To take any medications (excepted hormonal contraception, finasteride, benzodiazepine, Proton-pump inhibitors)
- To have a significant medical history (See Table: Prohibited conditions)
- Participation of another family member (first-second-third degree)
- Acute or allergic disease during the previous 2 weeks
- Temperature >38°C during the previous 2 weeks
- Dentist consultation during the previous 2 weeks
- Vaccination during the previous 2 weeks
- Steroids treatment during the previous 2 weeks
- Shift workers (chronic jet lag)

Immunophenotyping

For Geocode cohort

Immunophenotyping was done at the laboratory of Erasme hospital (LHUB-ULB) in a standardized approach. The immunophenotyping included neutrophil, lymphocyte, basophil, eosinophil, monocyte, T cell (CD3+), B cell (CD19+), NK cell (CD3-CD16+CD56+), CD4+ T cell, CD8+ T cell, CD4-CD8- T cell, and CD4+CD8+ T cell counts.

Briefly, immune cells were stained using a BD- Multitest 6-color TB: CD3 FITC, CD16+56 PE, CD45 PERCP-CY5.5, CD4 PE-CY7, CD19 APC, CD8 APC-CY7 (BD Biosciences). The BD-multitest was directly added to an EDTA tube containing whole blood. After incubation, red cells were lysed and stained immune cells were analysed by Navios flow cytometer (Beckman Coulter).

For Liège cohort

Immunophenotyping was done at the laboratory of GIGA. The immunophenotyping included neutrophils, lymphocytes, monocytes, eosinophils and basophils were automatically counted in whole blood with an Abbott Cell-Dyn 3700 hematology analyzer. Red blood cells were lyzed with a PharmLyse buffer (BD Biosciences) for 10 minutes at 4°C. After washing, cells were stained with anti-CD3-APC/Fire 750 (BioLegend), anti-CD4-BV711 (BD Biosciences), anti-CD8-BV786 (BD Biosciences), anti-CD19-BB700 (BD Biosciences), anti-CD56-BV650 (BD Biosciences) antibodies and fixable viability dye 455UV (eBioscience) for 30 minutes at 4°C. Cells were then fixed with PBS containing 1% formaldehyde (Aldrich) and filtered using a 100 µm CellTrics filter (Sysmex). Data acquisition was performed on a FACS Fortessa instrument (BD Biosciences) which was calibrated using CS&T beads (BD Biosciences). Fluorescence compensations were performed using CompBeads (BD Biosciences). Data analysis was performed with FlowJo version 10 (Treestar). After exclusion of debris, doublets and dead cells, CD4+ and CD8+ T cells, B cells and NK cells were targeted.

Clustering and Factor-driven clustering

The GEOCODE dataset comprised initially 417 individuals represented by 12 features, whereas the LIEGE dataset was made of 214 individuals described by 10 features.

Individuals with missing entries were removed from the clustering analyses. In that way, 18 individuals were removed from the GEOCODE dataset, so that 389 subjects were finally considered for the clustering study, whereas 3 individuals were removed from the LIEGE dataset, with a final dataset size of 208 subjects. Feature scaling (i.e. subtracting each feature by its mean and dividing by its standard deviation) was also applied prior to the clustering procedure, in order to ensure that all features are equally considered.

Three clustering methodologies, representing the main families of clustering algorithms, have been employed for revealing the dataset structure: *Partition Around Medoids (PAM)* [1] (which belongs to the family of partitional clustering algorithms), *DBSCAN* [2] (for density-based clustering) and *Gaussian Mixture Model (GMM) clustering* [3] (for model-based clustering). These three complementary approaches were chosen to strengthen the confidence in the obtained results. Briefly, partitional clustering algorithms structure the input space by comparing data objects using a dedicated distance measure, and more particularly by assigning each data object to the cluster with the closest centroid or prototype (i.e., an object which is representative of the considered cluster). In the case of the PAM algorithm, the prototypes are the cluster medoids, i.e. the objects which minimize the sum of distances with all the considered cluster objects. In this work, PAM was used in combination with the Euclidean distance. While PAM represents a well-known algorithm with some interesting properties (such as, e.g., a better robustness to outliers and noise compared to the traditional K-means algorithm [4]), it cannot find non-convex clusters. Also, the number of clusters has to be fixed a priori. To alleviate limits related to PAM, density-based clustering, and more particularly the DBSCAN technique, has also been employed. The main idea consists in partitioning the input space by separating dense from sparse zones. The algorithm is in that way able to detect clusters of arbitrary shapes. Finally, Gaussian Mixture Model clustering, belonging to the family of model-based clustering techniques, was employed in order to ascertain our results. GMM clustering aims indeed at discovering the structure by fitting the dataset to multi gaussian distributions using Maximum Likelihood Estimation (MLE), and is thus able to discover overlapping clusters.

The two datasets were then augmented by adding intrinsic as well as environmental factors to the initial 12 features (10 for the LIEGE cohort). Five factors were in that way appended to the GEOCODE cohort, namely Gender (Male/Female), Age (<29 years old, >=29 years old), Body Mass Index (BMI) (<20, [20-25], >25), EBV (1 in case of a positive EBV test and 0 otherwise) and CMV (1 in case of a positive CMV test and 0 otherwise). Three factors were added to the LIEGE cohort dataset: namely Gender (Male/Female), Age (<51 years old, >=51 years old) and BMI (<20, [20-25], >25).

By doing so, we aim at revealing structure in the dataset which could be possibly induced by the consideration of such factors.
A factor-driven clustering framework, which consists in clustering the datasets according to the predefined values of the studied intrinsic/environmental factors, was then proposed. we visually assessed the clustering tendency of a dataset by applying a Principal Component Analysis (PCA) [12] and t-distributed stochastic neighbour embedding (tSNE) [13], and we tagged the dataset according to the predefined values of the studied intrinsic/environmental factors. Partitions were generated automatically for each combination of factor values. Partitions were generated automatically for each combination of factor values. A mono-factor analysis, in which each factor was considered separately, has first been performed, followed by a multi-factor analysis in which all possible combinations were tested. In total, 31 combinations were finally investigated to unveil the presence of substructure in the GEOCODE cohort, and 7 configurations for the LIEGE cohort.

Models calibration and evaluation methods
Evaluating partitions obtained by clustering approaches faces two main challenges. First, the ground truth is usually non-accessible to assess the quality of a partition, so that the 'true' number of clusters is unknown in practice. Second, the complete absence of structure in a dataset, which corresponds to a situation where a single cluster solution is the most suited, is not easily quantified by common clustering evaluation methods, especially in the case of partitional algorithms. To deal with these two challenges, three different evaluation metrics were employed for assessing the quality of the solution given by the partitional clustering algorithms (the PAM algorithm in this case) and infer the correct number of clusters. On the other hand, an ad-hoc evaluation criterion was provided for the DBSCAN and GMM clustering techniques, since these approaches are inherently able to suggest the best number of clusters, as explained below. These quantitative techniques were completed by a qualitative evaluation criterion based on a visual analysis of the input dataset.
Three evaluation methods were employed to evaluate the results obtained by PAM, i.e., the *Silhouette Index*, the *Gap Statistic*, as well as a Cluster Stability Analysis. The *Silhouette index* [1] evaluates jointly the separability and the cohesion of the clustering solution, and provides local (an index for each object) as well as global (average of all local silhouettes) indices, comprised between -1 and 1 (the closest to 1 the better, a negative index indicates wrong cluster assignment. The Silhouette Index has been selected for its good performance on datasets from various scientific fields [5]. However, it is not defined when the number of clusters is equal to 1, and is thus unable to firmly confirm the possible absence of structure in a given dataset. The Gap Statistic [6] compares the clustering solution obtained from the studied dataset with partitions computed from multiple unstructured (or 'single component') datasets generated artificially. It can thereby detect single clustered datasets. Finally, Cluster Stability analysis [7] relies on the fact that a good partition should remain stable with respect to perturbations. To that end, partitions obtained on random subsamples of the original dataset were compared on the basis of a similarity score, in our case the Jaccard index [8].

These scores were represented in histograms, for different numbers of clusters: histograms with a narrow dispersion correspond to stable partitions, whereas unstable solutions are associated with wide histograms.
The DBSCAN algorithm is a density-based clustering technique which directly suggests the ideal number of clusters when applied on a given dataset, and is able to identify outliers and noise. However, the outcomes of the algorithm depend on the values taken by two parameters, namely ε, a distance which defines the neighbourhood of a given data object x (or ε-neighbourhood), and *minPts*, the minimum number of points which should be comprised in the ε-neighbourhood of x to consider that they belong to the same cluster. In this work, the optimal value of ε was chosen (1) by computing the nearest-neighbour distances of each data object, (2) by sorting them in ascending order, and (3) by selecting the distance which corresponds to a knee in the ordered distance curve [2]. *minPts* was chosen to be successively equal to the dimensionality of the dataset + 1 (i.e. 13 in the GEOCODE dataset), and to the first multiples of the dataset dimensionality (i.e. 24, 36 and 48 for the GEOCODE cohort), which is standard practice in the data science community. More precisely, we have shown that, for the two cohorts, the different values chosen for *minPts* have not modified the obtained results.
In the case of model-based clustering using GMMs, the algorithm was run with an increasing number of clusters (or gaussian components), and the model which provides the best Bayesian Information Criterion (BIC), an index that quantifies to what extent the model correctly fits the input space data, was selected.

ADDITIONAL BIBLIOGRAPHY

1. Rousseeuw, P.J., *Silhouettes: A graphical aid to the interpretation and validation of cluster analysis.* Journal of Computational and Applied Mathematics, 1987. **20**: p. 53-65.
2. Ester, M., et al., *A density-based algorithm for discovering clusters in large spatial databases with noise*, in *Proceedings of the Second International Conference on Knowledge Discovery and Data Mining*. 1996, AAAI Press: Portland, Oregon. p. 226–231.
3. Melnykov, V. and R. Maitra, *Finite mixture models and model-based clustering.* Statist. Surv., 2010. **4**: p. 80-116.
4. Van der Laan, M., K. Pollard, and J. Bryan, *A new partitioning around medoids algorithm.* Journal of Statistical Computation and Simulation, 2003. **73**(8): p. 575-584.
5. Arbelaitz, O., et al., *An extensive comparative study of cluster validity indices.* Pattern Recognition, 2013. **46**(1): p. 243-256.
6. Tibshirani, R., G. Walther, and T. Hastie, *Estimating the number of clusters in a data set via the gap statistic.* Journal of the Royal Statistical Society: Series B (Statistical Methodology), 2001. **63**(2): p. 411-423.
7. von Luxburg, U., *Clustering Stability: An Overview.* Foundations and Trends® in Machine Learning, 2010. **2**(3): p. 235-274.
8. Ben-Hur, A., A. Elisseeff, and I. Guyon, *A stability based method for discovering structure in clustered data.* Pac Symp Biocomput, 2002: p. 6-17.

SUPPLEMENTARY TABLE 1

	Female (n=123)	Male (n=85)	p-value
**Age**	51 (32-60)	52 (35-62)	0.35
**BMI**	23.7 (20.7-27.5)	26.3 (23.2-29.1)	**0.001**

**Neutrophils**	3290 (2665 – 4052)	3125 (2530 – 4087)	0.62
**Lymphocytes**	1810 (1550 – 2210)	1710 (1317– 2012)	**0.03**
**Basophiles**	46 (26- 62)	43 (26-65)	0.63
**Eosinophils**	112 (80-186)	116 (76-187)	0.72
**Monocytes**	402 (324-490)	439 (370-527)	**0.04**
**T cells**	1001 (720-1389)	829 (566-1181)	**0.02**
**CD4+ T cells**	751 (549-1016)	596 (427-836)	**0.006**
**CD8+ T cells**	181 (106-333)	150 (81-300)	0.18
**NK cells**	209 (116-318)	230 (115-407)	0.14
**B cells**	136 (94-177)	95 (70-182)	0.08

LEGEND OF SUPPLEMENTARY FIGURES

Supplementary Figure 1: Coefficient of variations regarding the immune cells in GEOCODE study cohort and in the replication cohort. Coefficient of variation (CV) was calculated as ratio between standard deviation and mean for each leukocyte subset. The CV ranged from 30% to 100% for all the features but basophiles in GEOCODE cohort.

Supplementary Figure 2: Clustering approaches applied to LIEGE replication cohort. **A.** Three-dimensional PCA of the study was used as a qualitative visual criterion. **B.** The graph shows the average silhouette indices obtained by running PAM with an increasing number of clusters *K*. For each value of *K*, 20 instances of the PAM algorithm were run with a random initialization of centroids, in order to mitigate the effect of local minima. The corresponding solutions are represented by black circles, whereas the best solutions for each *K*, i.e. the partitions which maximize the Silhouette Index, are connected by a plain red line. **C.** The graph shows the Gap Statistic as a function of the number of clusters. For each value of *K*, the Gap Statistic was computed by comparing the clustering solution obtained on the dataset with partitions extracted from 200 unstructured datasets randomly generated. **D.** The graph shows the results of the Cluster Stability Analysis, for which 100 random subsamples of the original dataset have been clustered and compared using the Jaccard Index.

Supplementary Figure 3: Gaussian Mixture Model clustering. the 2-dimensional plots show all the pairwise combinations of the input features for both datasets (blue dots), along with a representation of the single Gaussian distribution which best fits the data (in brown).

Supplementary Figure 4: Proportion of variance explained by intrinsic and extrinsic factors. Using linear regression model, the coefficient of determination (R^2) was calculated for each leukocyte subset and CRP. This coefficient reflects the proportion of the variance in the intrinsic and extrinsic factors which are predictable from the independent variables.

Supplementary figures

Supplementary Figure 1

GEOCODE study cohort

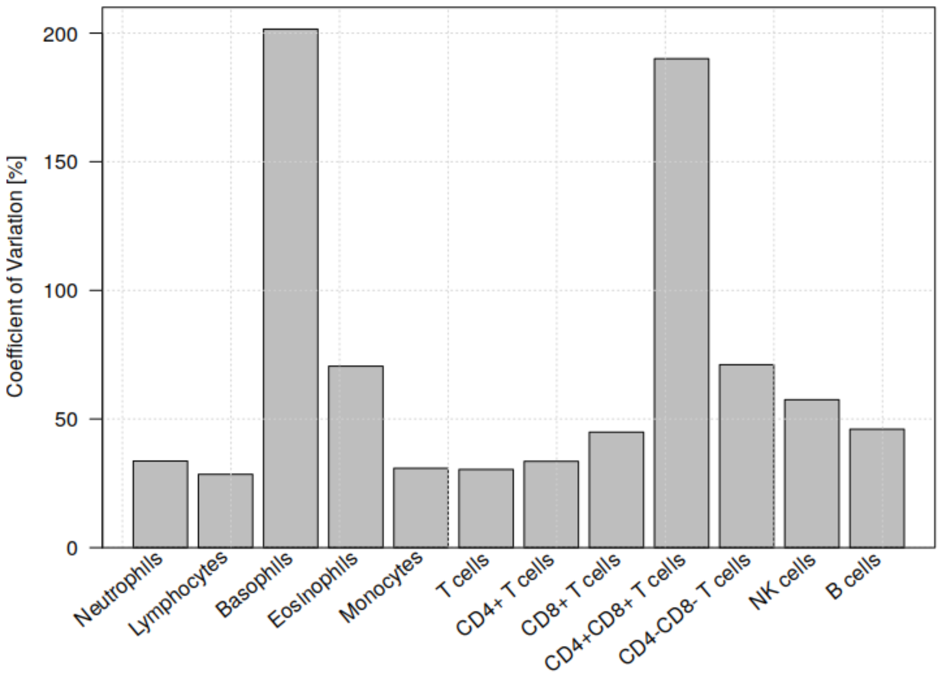

LIEGE replication cohort

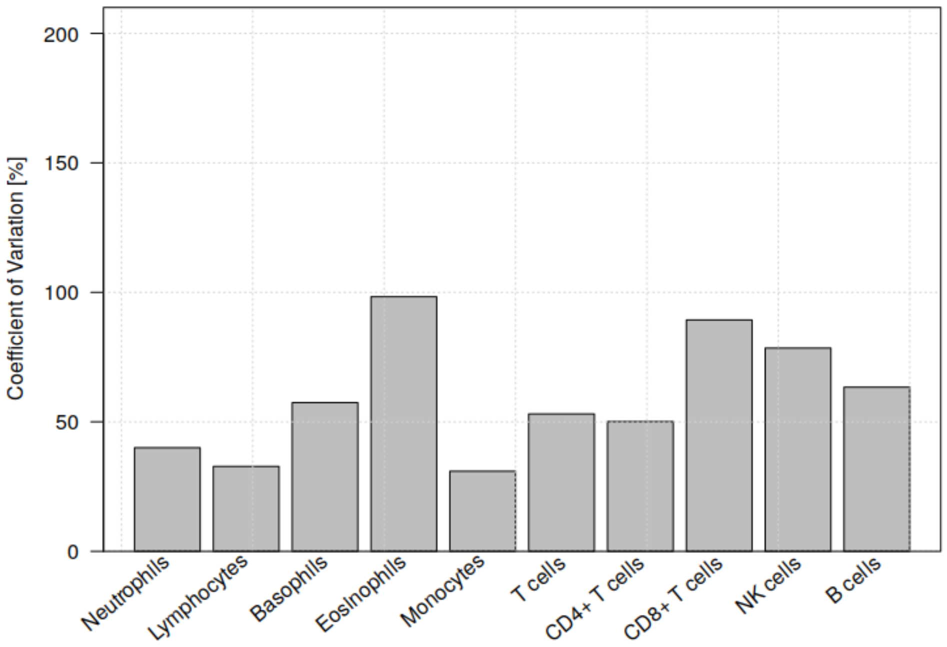

Supplementary Figure 2

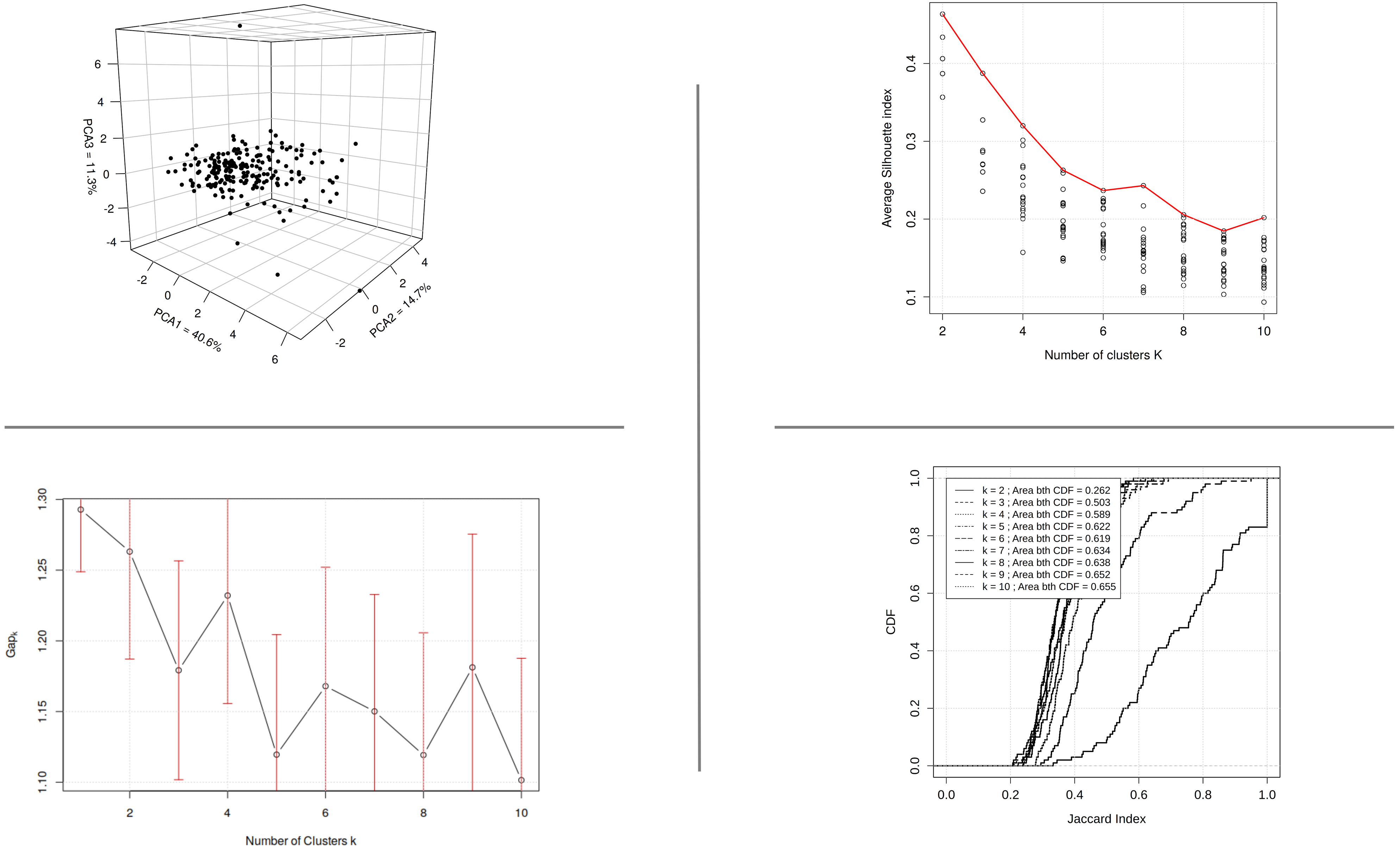

Supplementary Figure 3

GEOCODE study cohort

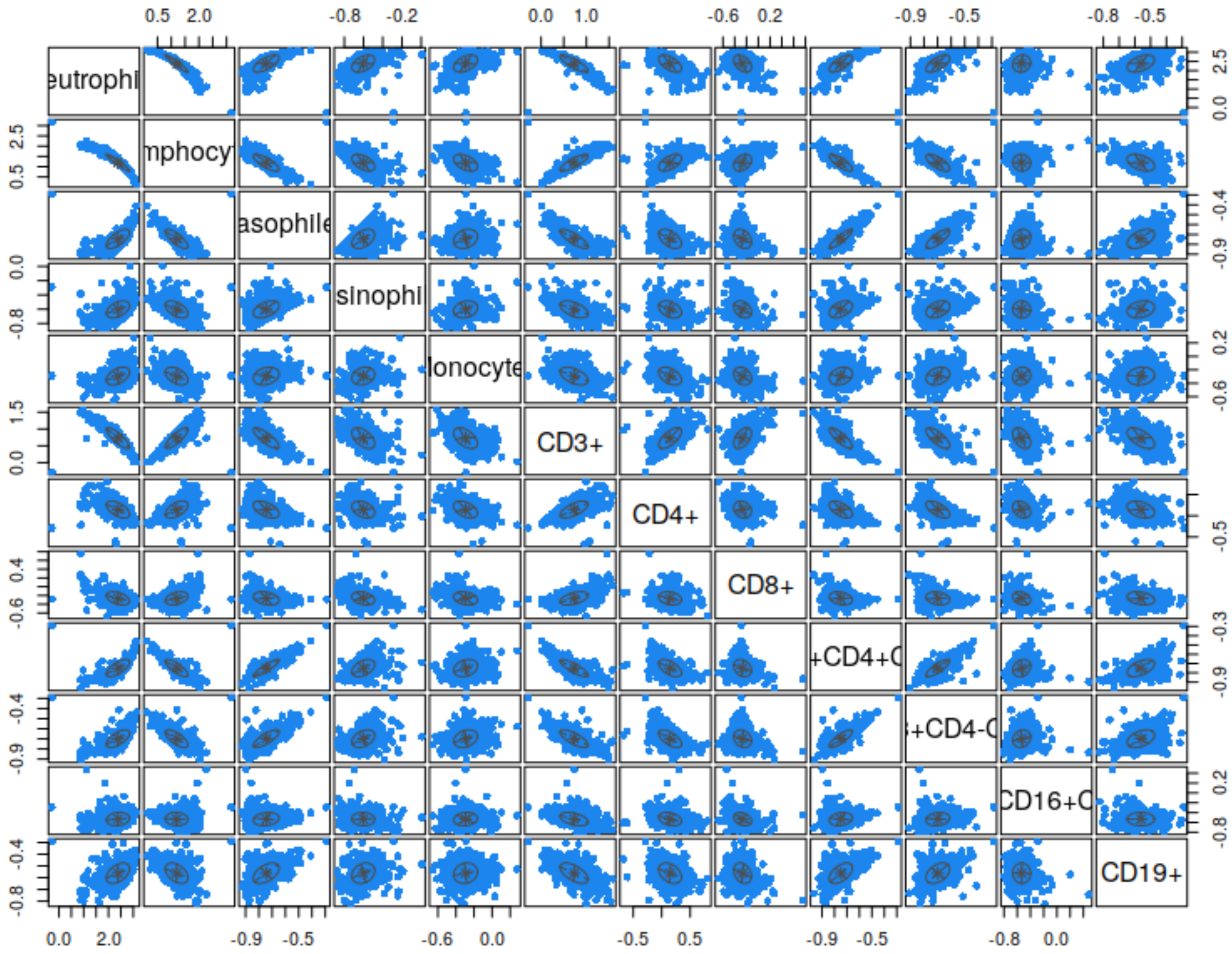

LIEGE replication cohort

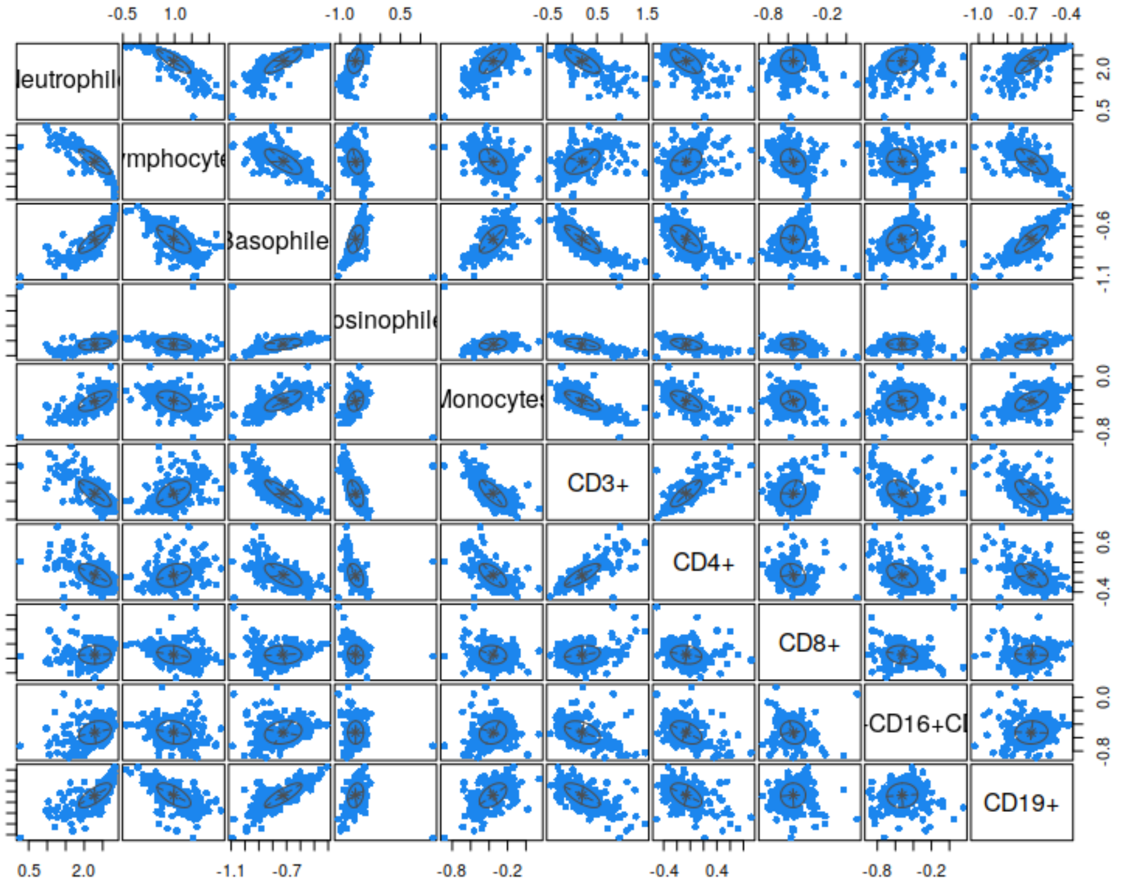

Supplementary Figure 4

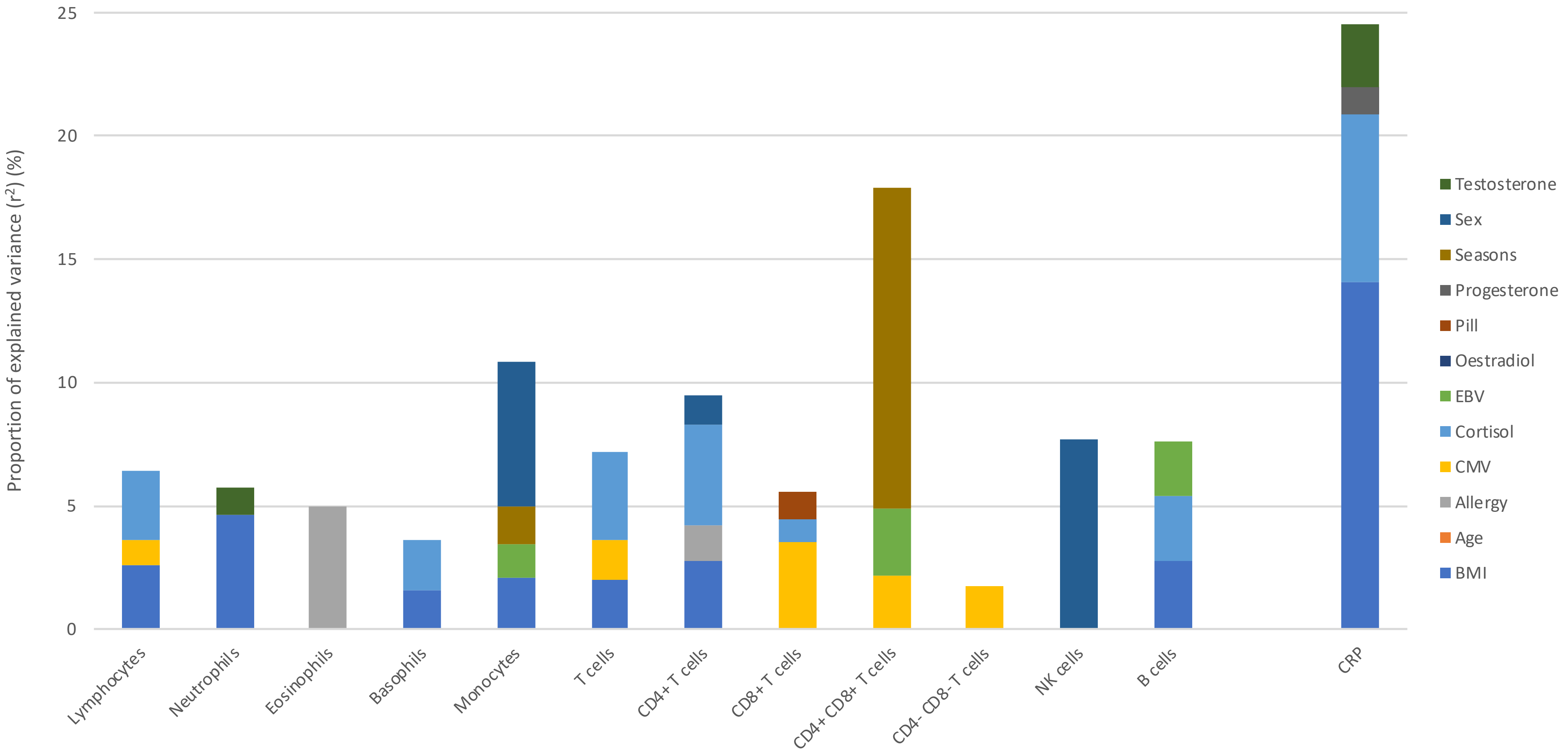

Supplement: Supplementary file 1 — Supplementary Information. [file 41598_2021_88272_MOESM1_ESM.pdf]
